# Evaluation of the retinal vasculature of patients with branch retinal vein occlusion using optical coherence tomography angiography

**DOI:** 10.5935/0004-2749.20200091

**Published:** 2024-02-11

**Authors:** Bárbara de Carvalho Freire Santos Moura, Rodrigo Pessoa Cavalcanti Lira, Tiago Eugênio Faria e Arantes, Isabel Braga Paiva, Vasco Torres Fernandes Bravo Filho

**Affiliations:** 1 Universidade Federal de Pernambuco, Recife, PE, Brazil; 2 Fundação Altino Ventura, Recife, PE, Brazil

**Keywords:** Retinal vein occlusion, Capillaries/pathology, Retinal vessels/physiopathology, Fovea centralis, Tomography, optical coherence, Fluorescein angiography, Oclusão de veia retiniana, Capilares/patologia, Vasos retinianos/fisiopatologia, Fóvea central, Tomografia de coerência óptica, Angiofluoresceinografia

## Abstract

**Purpose:**

To evaluate vascular density in super ficial and deep capillary plexuses of the retina, measured using optical coherence tomography angiography in patients with branch retinal vein occlusion. Affected eyes were compared with the contralateral eye of the same patient and both were compared with normal eyes.

**Methods:**

A cross-sectional study including 16 previously untreated patients with branch retinal vein occlusion. Patients with poor quality examinations, bilateral disease, high refractive error, or any other retinal or choroidal disease were excluded. A total of 31 patients without eye disease were also selected as a comparison group. All participants underwent five optical coherence tomography angiographies, and only those with at least two good quality examinations were selected. The Kruskal-Wallis, Wilcoxon signed-rank, and Mann-Whitney U tests were used for the statistical analysis.

**Results:**

Vascular density was lower in affected eyes compared with contralateral eyes: whole density (p=0.020 for capillary plexuses superficial; p=0.049 for deep capillary plexuses) and parafoveal density (p=0.020 for capillary plexuses superficial; p=0.011 for deep capillary plexuses). Vascular density was also lower in affected eyes compared with normal eyes: whole density (p<0.001 for capillary plexuses superficial and deep) and parafoveal density (p<0.001 for capillary plexuses superficial and deep). Whole density (p=0.001 for capillary plexuses superficial and deep) and parafoveal density (p=0.001 for capillary plexuses superficial; p<0.001 for deep capillary plexuses) were both lower in the contralateral eyes compared with normal eyes. Following adjustment for arterial hypertension, this difference was no longer observed.

**Conclusions:**

Vascular density in capillary plexuses and deep capillary plexuses was lower in the eyes affected by branch retinal vein occlusion. Furthermore, the lower vascular density noted in the contralateral eyes indicates that changes most likely occurred in these eyes prior to the appearance of any clinically detectable alterations, reflecting the early signs of hypertensive retinopathy.

## Abstract (pt)

**Objetivo:**

Avaliar a densidade vascular do plexo capilar superficial e profundo da retina, usando angiografia por tomografia de coerência óptica em pacientes com oclusão de ramo da veia central da retina, comparando o olho afetado com o contralateral do mesmo paciente e ambos com olhos normais.

**Métodos:**

Estudo transversal. Incluídos dezesseis pacientes com oclusão de ramo da veia central da retina sem tratamento prévio. Pacientes com exames de baixa qualidade, altas ametropias, outras patologias de retina ou coróide foram excluídos. Para comparação, trinta e um pacientes sem doença ocular foram selecionados. Todos foram submetidos a cinco exames angiografia por tomografia de coerência óptica, apenas aqueles com pelo menos dois exames de boa qualidade permaneceram no estudo. Os testes Kruskal-Wallis, Wilcoxon, e Mann-Whitney foram utilizados.

**Resultados:**

Densidades vasculares mais baixas do plexo capilar superficial e plexo capilar profundo foram observadas quando olhos com oclusão de ramo da veia central da retina foram comparados com os contralaterais: densidade total (p=0,02 para plexo capilar superficial, p=0,049 para plexo capilar profundo), densidade parafoveal (p=0,02 para plexo capilar superficial, p=0,011 para plexo capilar profundo). Comparando olhos acometidos com olhos normais, também foram observadas densidades vasculares mais baixas de plexo capilar superficial e plexo capilar profundo: densidade total (ambos com p<0,001) e densidade parafoveal (ambos com p<0,001). Quando os olhos contralaterais foram comparados aos normais, tanto a densidade total do plexo capilar superficial e plexo capilar profundo (ambos com p=0,001) quanto a densidade parafoveal (plexo capilar superficial com p=0,001, plexo capilar profundo com p<0,001) foram menores. Ao se realizar uma subanálise, minimizando o fator hipertensão arterial, esta diferença não se manteve.

**Conclusões:**

Densidades vasculares mais baixas do plexo capilar superficial e do plexo capilar profundo foram observadas em olhos com oclusão de ramo da veia central da retina. Além disso, a presença de densidades vasculares mais baixas nos olhos contralaterais mostra que já existem altera ções nesses olhos antes das alterações clínicas, devido a al terações inicias da retinopatia hipertensiva.

## INTRODUCTION

Retinal vein occlusion is the second most common retinal vascular disease^(^1^,^2^)^. In branch retinal vein occlusion (BRVO), blockage occurs in a branch of the retinal veins alone^(^3^-^5^)^. Visual prognosis depends on the presence of macular edema^(^6^,^7^)^ and the extent of the areas of non-perfusion^(^3^,^8^)^.

Until recently, the only available method for the evaluation of the areas of non-perfusion was through the use of fluorescein angiography^(^9^,^10^)^; however, this is an invasive procedure that uses intravenous contrast and associated with potential side effects^(^11^,^12^)^. Optical coherence tomography (OCT) angiography is a non-invasive, non-contact method that allows the three-dimensional in vivo visualization of the cross-sections of the retina and its vascular layers^(^13^-^16^)^.

The AngioAnalytics software (AngioVue RTVue XR OCT; Optovue Inc., Fremont, CA, USA) uses the algorithm referred to as split-spectrum amplitude-decorrelation angiography, which detects the movement of erythrocytes between two consecutive B-scan cross-sectional images, allowing the measurement of the vascular density of the superficial (SCP) and deep capillary plexuses (DCP) of the retina^(^14^,^17^-^19^)^. These parameters may be useful for evaluating damage to the retinal vasculature caused by BRVO. Moreover, they can be used to determine whether vascular density is also lower in the contralateral eye, which appears normal at clinical examination in patients affected by this disease. Evidence suggests that the contralateral eyes of patients with retinal vein occlusion are at a greater risk of occlusive episodes compared with those of the general population, since they are exposed to the same risk factors^(^8^)^.

The present study was designed to evaluate the parameters of vascular density of the SCP and DCP in cases with BRVO, by comparing the BRVO-affected eye with the contralateral eye of the same patient. This was subsequently followed by a comparison of both eyes with normal eyes of individuals in a control group.

## METHODS

This observational, cross-sectional study included a group of patients with BRVO and a control group consisting of individuals with healthy eyes. The study was performed in compliance with the tenets of the 1964 Declaration of Helsinki. The objectives and possible consequences of the study were explained to all participants, who subsequently provided written informed consent. The internal review board of the Altino Ventura Foundation (Recife, Brazil) approved the study protocol.

The study was conducted at both the Fundação Altino Ventura and the Hospital de Olhos de Pernambuco, both in Recife, Brazil, between October 2016 and May 2017. An active search of the medical records of untreated patients who had been diagnosed with BRVO at these institutes from January 2016 onwards was conducted. These patients were contacted and requested to undergo a complete ophthalmologic examination in the aforementioned institutes. This included evaluation of their best-corrected visual acuity assessed using a logarithm of the minimum angle of resolution (logMAR) vision chart according to the Early Treatment Diabetic Retinopathy Study testing protocol. In addition, patients were subjected to biomicroscopy of the anterior and posterior segments of the eye, fundoscopy performed using a Volk Superfield lens (Volk Optical Inc., Mentor, OH, USA), tonometry using a Goldmann tonometer, and gonios copy using a Volk G6 gonio lens. Examination was performed prior to OCT angiography to rule out the presence of any retinal or choroidal pathologies.

Eligible participants were patients diagnosed with BRVO who had not undergone any previous treatment. Patients with any sign of diabetic retinopathy, retinal dystrophies or degeneration, myopia above 6D, or hyperopia above 6D were excluded. In addition, OCT angiography with poor quality images involving artifacts, signal strength index (SSI) <45, or segmentation errors constituted another exclusion criterion. The contralateral eyes of the same patients were used as a comparative group, based on the same exclusion criteria. Patients with bilateral disease were excluded from the study. The eyes of patients were also compared with those of a group of individuals without ophthalmologic disease and best-corrected visual acuity >0.1 logMAR. The same exclusion criteria were applied to this control group, with the addition of the presence of hypertensive retinopathy.

The participants were subjected to spectral domain OCT (AngioVue RTVue XR Avanti spectral-domain OCT; Optovue Inc.), using the AngioAnalytics software. A scan capture area of 6 × 6 mm centered on the fovea was used. Each B-scan consisted of 304 A-scans. A total of 304 B-scans were performed, involving two repeated B-scans, captured for 2.6 s at each fixed position and then proceeding to the next sampling location. A single trained operator captured five OCT angiography images of each eye. Only participants with at least two good quality examinations, (i.e., SSI >45 and absence of motion artifacts or segmentation errors) remained in the study. The density parameters SCP and DCP of each patient were determined from the vessel density map provided by the AngioAnalytics software, with mean values being used in the analysis. The SCP was considered to be at a depth of 3 µm below the internal limiting membrane at 15 µm below the internal border of the inner plexiform layer. The DCP was considered to be at a depth of 15-70 µm below the inner plexiform layer. The SPSS version 24.0 for Windows (IBM Corp., Armonk, NY, USA) was used for the statistical analysis. The quantitative variables are expressed as the means and standard deviations, while the qualitative variables are expressed as absolute and relative frequencies. The Wilcoxon signed-rank test and the Mann-Whitney U test were used in the analysis. P-values <0.05 denoted statistically significant differences.

## RESULTS

A total of 30 patients diagnosed with BRVO were subjected to OCT angiography, with five images being captured for each case. Only those with at least two good quality examinations remained in the study; 14 patients were excluded from this group. In the control group, 31 participants with normal eyes were subjected to OCT angiography and five images were captured for each case. When both eyes were found to be normal, one eye was randomly selected for inclusion in the study. There was no exclusion of participants in this group.

The characteristics of the participants included in the study (e.g., age, sex, duration of the disease, and certain risk factors) are summarized in table 1. The two groups were similar, except for the significantly higher percentage of individuals in the BRVO group who had risk factors (e.g., systemic arterial hypertension, a history of thrombosis, cardiovascular disease, and smoking). The mean duration of the disease was 6.4 ± 4.8 months (range: 1-17 months).

**Table 1:** Sociodemographic characteristics and risk factors of the participants

	Control (n=31)	Branch retinal vein occlusion (n=16)	p-value^*^
Age (years), mean ± SD (range)	51.3 ± 13.4 (23-78)	52.1 ± 9.7 (33-66)	0.770
Duration of disease (months), mean ± SD (range)	--	6.4 ± 4.8 (1-17)	--
Sex, n (%) Male	9 (29)	6 (37.5)	0.555
Female	22 (71)	10 (62.5)	
Systemic arterial hypertension, n (%) No	25 (80.6)	3 (18.8)	<0.001
Yes	6 (19.4)	13 (81.3)	
Diabetes mellitus, n (%) No	28 (90.3)	13 (81.3)	0.395
Yes	3 (9.7)	3 (18.8)	
Smoking, n (%)		.	
No	29 (93.5)	11 (68.8)	0.036
Yes	2 (6.5)	5 (31.3)	
Glaucoma, n (%) No	28 (90.3)	12 (75.0)	0.208
Yes	3 (9.7)	4 (25.0)	
Cardiovascular disease, n (%) No	30 (96.8)	12 (75.0)	0.040
Yes	1 (3.2)	4 (25.0)	
History of thrombosis, n (%) No	31 (100)	13 (81.3)	0.035
Yes	0 (0)	3 (18.8)	
Oral contraceptive use (females), n (%)^†^ No	20 (90.9)	9 (90.0)	<0.999
Yes	2 (9.1)	1 (10.0)	

* = The Mann-Whitney U test was used to compare age between the two groups and the chi-squared test to compare the categorical variables.

^†^= Data for oral contraceptive use by the female participants refer to 22 females evaluated in the control group and 10 females in the branch retinal vein occlusion group. SD= standard deviation.

Mean SSI was 58.8 ± 7.8 (range: 46.0-69.6) for the affected eyes in the BRVO group compared with 66.3 ± 7.8 (range: 51.8-78.4) for the contralateral eyes and 73.8 ± 6.2 (range: 59.0-81.0) for the normal eyes in the control group (Tables 2 and 3).

**Table 2:** Comparison between the branch retinal vein occlusion (BRVO)-affected eyes and the contralateral eyes

	BRVO-affected eyes (n=16)		Contralateral eyes (n=16)		
	**Mean ± SD**	**Range**	**Mean ± SD**	**Range**	p-value^*^
Signal strength index	58.8 ± 7.8	46.0-69.6	66.3 ± 7.8	51.8-78.4	0.005
Whole density (superficial)	46.4 ± 4.2%	38.5-52.3	49.1 ± 3.9%	41.7-54.7	0.020
Foveal density (superficial)	28.0 ± 5.6%	19.6-40.6	25.4 ± 5.7%	15.3-32.7	0.326
Parafoveal density (superficial)	47.1 ± 5.0%	38.6-54.6	51.0 ± 5.6%	42.1-58.1	0.020
Whole density (deep)	47.8 ± 4.6%	38.6-54.4	50.4 ± 4.2%	42.4-57.6	0.049
Foveal density (deep)	29.4 ± 6.3%	18.7-40.8	24.7 ± 5.6%	15.4-33.1	0.098
Parafoveal density (deep)	48.8 ± 5.4%	38.7-57.2	53.1 ± 5.8%	43.0-61.3	0.011

* = Wilcoxon signed-rank test; SD= standard deviation.

**Table 3:** Comparison between branch retinal vein occlusion (BRVO)-affected eyes and normal eyes

	BRVO-affected eyes (n=16)		Normal eyes (n=31)		p-value^*^
	Mean ± SD	Range	Mean ± SD	Range	
Signal strength index	58.8 ± 7.8	46.0-69.6	73.8 ± 6.2	59.0-81.0	<0.001
Whole density (superficial)	46.4 ± 4.2%	38.5-52.3	53.0 ± 3.4%	43.7-57.9	<0.001
Foveal density (superficial)	28.0 ± 5.6%	19.6-40.6	29.4 ± 6.1%	19.5-41.5	0.419
Parafoveal density (superficial)	47.1 ± 5.0%	38.6-54.6	56.3 ± 3.8%	46.2-61.9	<0.001
Whole density (deep)	47.8 ± 4.6%	38.6-54.4	54.6 ± 3.6%	43.7-59.8	<0.001
Foveal density (deep)	29.4 ± 6.3%	18.7-40.8	29.6 ± 7.0%	18.8-46.4	0.928
Parafoveal density (deep)	48.8 ± 5.4%	38.7-57.2	58.6 ± 4.0%	47.4-64.5	<0.001

* = Mann-Whitney U test; SD= standard deviation.

Vascular density was lower in the eyes affected by BRVO versus the contralateral eyes both with respect to whole density (p=0.02 for SCP; p=0.049 for DCP) and parafoveal density (p=0.02 for SCP; p=0.011 for DCP) (Table 2). Similar findings were recorded when BRVO-affected eyes were compared with the normal eyes of individuals in the control group, revealing lower whole density (p<0.001 for both SCP and DCP) and parafoveal density (p<0.001 for SCP and DCP) (Table 3).

Both the whole density of the SCP and DCP (p=0.001 in both cases) and parafoveal density of the SCP and DCP (SCP: p=0.001; DCP: p<0.001) were lower in the contralateral eyes of the individuals affected by BRVO compared with the normal eyes (Table 4).

**Table 4:** Comparison between the contralateral eyes of patients with branch retinal vein occlusion and normal eyes

	Contralateral eyes (n=16)		Normal eyes (n=31)		
	**Mean ± SD**	**Range**	**Mean ± SD**	**Range**	p-value^*^
Signal strength index	66.3 ± 7.8	51.8-78.4	73.8 ± 6.2	59.0-81.0	0.001
Whole density (superficial)	49.1 ± 3.9%	41.7-54.7	53.0 ± 3.4%	43.7-57.9	0.001
Foveal density (superficial)	25.4 ± 5.7%	15.3-32.7	29.4 ± 6.1%	19.5-41.5	0.076
Parafoveal density (superficial)	51.0 ± 5.6%	42.1-58.1	56.3 ± 3.8%	46.2-61.9	0.001
Whole density (deep)	50.4 ± 4.2%	42.4-57.6	54.6 ± 3.6%	43.7-59.8	0.001
Foveal density (deep)	24.7 ± 5.6%	15.4-33.1	29.6 ± 7.0%	18.8-46.4	0.037
Parafoveal density (deep)	53.1 ± 5.8%	43.0-61.3	58.6 ± 4.0%	47.4-64.5	<0.001

* = Mann-Whitney U test; SD= standard deviation.

There was no statistically significant difference in the foveal density of the SCP and DCP when the BRVO-affected eyes were compared with the contralateral or normal eyes or when the contralateral eyes were compared with the normal eyes (Tables 2-4).

In the group of normal eyes, all hypertensive patients and one patient with normal blood pressure were selected to balance the proportions of this disorder in both groups (Table 5), in an attempt to cancel out the imbalance in systemic arterial hypertension. Comparison between the affected and normal eyes in this subgroup showed that both the whole density and parafoveal density of the SCP were statistically lower in the affected eyes (p=0.042 and p=0.013, respectively). With respect to the DCP, only the parafoveal density was significantly lower (p=0.011). There was no statistically significant difference in the foveal density (Table 6).

**Table 5:** Sociodemographic characteristics and risk factors of the participants, following adjustment for systemic arterial hypertension (SAH)

	Control (after adjustment for SAH) (n=7)	Branch retinal vein occlusion (n=16)	p-value^*^
Age (years), mean ± SD (range)	59.3 ± 11.0 (48-74)	52.1 ± 9.7 (33-66)	0.216
Duration of disease (months), mean ± SD (range)	--	6.4 ± 4.8 (1-17)	--
Sex, n (%)			>0.99
Male	2 (28.6)	6 (37.5)	
Female	5 (71.4)	10 (62.5)	
Systemic arterial hypertension, n (%)			>0.99
No	1 (14.3)	3 (18.8)	
Yes	6 (85.7)	13 (81.3)	
Diabetes mellitus, n (%)			0.62
No	5 (71.4)	13 (81.3)	
Yes	2 (28.6%)	3 (18.8)	
Smoking, n (%)		.	0.27
No	7 (100)	11 (68.8)	
Yes	0 (0)	5 (31.3)	
Glaucoma, n (%)			>0.99
No	5 (71.4)	12 (75.0)	
Yes	2 (28.6)	4 (25.0)	
Cardiovascular disease, n (%)			>0.99
No	6 (85.7)	12 (75.0)	
Yes	1 (14.3)	4 (25.0)	
History of thrombosis, n (%)			0.53
No	7 (100)	13 (81.3)	
Yes	0 (0)	3 (18.8)	
Oral contraceptive use (females), n (%)^†^			>0.99
No	5 (100)	9 (90.0)	
Yes	0 (0)	1 (10.0)	

* = The Mann-Whitney U test was used to compare age between the two groups and the chi-squared test to compare the categorical variables.

† = Data for oral contraceptive use by the female participants refer to five females evaluated in the control group and 10 females in the branch retinal vein occlusion group. SD= standard deviation.

**Table 6:** Comparison between branch retinal vein occlusion (BRVO)-affected eyes and normal eyes, following adjustment for systemic arterial hypertension (SAH)

	BRVO-affected eyes (n=16)		Control (after adjustment for SAH)		
	**Mean ± SD**	**Range**	**Mean ± SD**	**Range**	p-value^*^
Signal strength index	58.8 ± 7.8	46.0-69.6	71.1 ± 6.6	60.0-79.0	0.004
Whole density (superficial)	46.4 ± 4.2%	38.5-52.3	50.4 ± 3.0%	47.4-54.9	0.042
Foveal density (superficial)	28.0 ± 5.6%	19.6-40.6	27.1 ± 4.3%	20.4-31.7	<0.99
Parafoveal density (superficial)	47.1 ± 5.0%	38.6-54.6	53.5 ± 4.2%	47.3-58.7	0.013
Whole density (deep)	47.8 ± 4.6%	38.6-54.4	51.8 ± 2.8%	48.2-55.5	0.061
Foveal density (deep)	29.4 ± 6.3%	18.7-40.8	27.3 ± 4.7%	20.1-32.2	0.442
Parafoveal density (deep)	48.8 ± 5.4%	38.7-57.2	55.4 ± 4.2%	48.9-59.7	0.011

* = Mann-Whitney U test; SD= standard deviation.

Comparison of the contralateral eyes with the normal eyes in this subgroup did not reveal a statistically significant difference in any of the investigated parameters (Table 7).

**Table 7:** Comparison between the contralateral eyes of patients with branch retinal vein occlusion and normal eyes, following adjustment for systemic arterial hypertension (SAH)

	Contralateral eyes (n=16)		Control (after adjustment for SAH)		
	**Mean ± SD**	**Range**	**Mean ± SD**	**Range**	p-value^*^
**Signal strength index**	66.3 ± 7.8	51.8-78.4	71.1 ± 6.6	60.0-79.0	0.132
**Whole density (superficial)**	49.1 ± 3.9%	41.7-54.7	50.4 ± 3.0%	47.4-54.9	0.316
**Foveal density (superficial)**	25.4 ± 5.7%	15.3-32.7	27.1 ± 4.3%	20.4-31.7	0.593
**Parafoveal density (superficial)**	51.0 ± 5.6%	42.1-58.1	53.5 ± 4.2%	47.3-58.7	0.385
**Whole density (deep)**	50.4 ± 4.2%	42.4-57.6	51.8 ± 2.8%	48.2-55.5	0.504
**Foveal density (deep)**	24.7 ± 5.6%	15.4-33.1	27.3 ± 4.7%	20.1-32.2	0.285
**Parafoveal density (deep)**	53.1 ± 5.8%	43.0-61.3	55.4 ± 4.2%	48.9-59.7	0.242

* = Mann-Whitney U test; SD= standard deviation.

## DISCUSSION

This was the first study in which OCT angiography was used to evaluate previously untreated patients with BRVO. Patients diagnosed <17 months previously, who had not received any form of treatment, were selected. This approach allowed the assessment to be performed during the initial care of patients affected by BRVO irrespective of the presence or absence of macular edema, which is commonly encountered in this pathology.

Cystoid macular edema may hamper the visualization of the vascular plexuses and obstruct the detailed visualization of other retinal structures. Moreover, it produces artifacts and segmentation errors that ought to be considered when deciding whether the density map generated by the AngioAnalytics software is reliable. In addition, visual acuity was poor in many of these patients, hampering foveal fixation, and consequently hindering OCT angiography. Indeed, the investigators obtained a lower mean SSI in the group of BRVO-affected eyes than in that of normal eyes. In addition, 14 of the 30 patients with BRVO were excluded from the group of affected eyes, whereas none of the individuals in the control group of normal eyes were excluded. Five OCT angiography images were captured and only those patients with at least two reliable examinations remained in the study to ensure the quality of images. Three examiners evaluated each individual examination, including an experienced retinologist who ensured that only good quality images were included in the study.

Macular edema was present and absent in nine and seven of the patients with BRVO analyzed in the study. This finding showed that the presence of macular edema does not necessarily indicate that the parameters are unreliable, as long as the quality of the image is evaluated adequately.

Currently, OCT angiography is becoming increasingly important in the evaluation of retinal vascular diseases, including BRVO. The reduction in vascular perfusion has been shown to be greater in the deep vascular plexuses of the retina^(^13^,^17^,^20^,^21^)^. This fact limits the use of fluorescein angiography, which only provides a two-dimensional assessment. Other investigators have shown the foveal avascular zone to be significantly wider in occluded eyes compared with normal eyes. This increase may also be indicative of greater macular ischemia^(^13^,^21^-^24^)^.

Thus far, few studies using the new OCT angiography and density map have been conducted. The mean whole vascular density of 53.0 ± 3.4% recorded in the group of normal eyes in the present study was similar to that reported in previous studies (52.58 ± 3.22%^(^25^)^ and 53.6%^(^26^)^).

The primary objective of the present study was to assess whether this examination could be useful for quantifying the damage to the retinal vasculature caused by BRVO by measuring retinal density. Of note, a reduction in this density is directly associated with the degree of ischemia in these eyes. A significant correlation was previously identified between peripheral retinal nonperfusion and whole vascular density of the SCP and DCP^(^19^)^. Further studies may establish reference values for the diagnosis and follow-up of these patients. In the present study, the whole density was 46.4 ± 4.2% and 47.8 ± 4.6% for the SCP and DCP, respectively. The parafoveal density was 47.1 ± 5.0% and 48.8± 5.4%, respectively. Other studies have reported similar findings, with values of whole vascular density of 44.14 ± 4.91%^(^19^)^ and 48.07% (46.21%-49.92%)^(^27^)^ for the SCP, and 48.98 ± 4.97 %^(^21^)^ and 52.60% (50.82%-54.37%)^(^27^)^ for the DCP. The parafoveal density was 45.92 ± 5.26% for the SCP and 50.54 ± 5.38% for the DCP^(^19^)^.

Comparison of the group of eyes affected by BRVO with those of the contralateral eyes and normal eyes revealed lower whole density and parafoveal density of the SCP and DCP in the BRVO group. Other researchers have reported similar findings for both the whole density and parafoveal density of the SCP and DCP^(^19^)^ and for the whole density of the SCP and DCP alone^(^27^)^. In one of those studies^(^27^)^, the affected eye was compared with the contralateral eye but not with a normal eye. However, those investigators compared the affected sector with an unaffected sector, since this disease affects only part of the eye. The results yielded lower values in the affected region compared with the unaffected segment.

Another investigator also compared the parafoveal density of the SCP and DCP of BRVO-affected eyes with those of the contralateral eyes and normal eyes. However, that study evaluated retinal vein occlusions as a group, without distinguishing between BRVO and central retinal vein occlusion. Nonetheless, the density was also reported to be lower in the affected eyes^(^23^)^.

There was no statistically significant difference between the foveal density of the SCP and DCP of the retina in the present study. These results are in agreement with those of previous studies^(^19^,^24^,^27^)^. This is mainly attributed to the fact that foveal density encompasses the foveal avascular zone, and the density of the veins in this region is low.

There is evidence indicating that the contralateral eyes of patients with retinal vein occlusion are at a grea ter risk of an occlusive event compared with those of the general population, since both eyes are submitted to the same risk factors^(^8^)^. In the present study, the whole density and parafoveal density of the SCP in the contralateral eyes of the patients with BRVO were 49.1 ± 3.9% and 51.0 ± 5.6%, respectively. The values for the DCP were 50.4 ± 4.2% and 53.1 ± 5.8%, respectively. These values were significantly lower than those found in the group of normal eyes. There was no statistically significant difference between the foveal densities of the contralateral eyes and those of the normal eyes.

Another study also reported alterations detected by OCT angiography in the contralateral eyes of such patients^(^13^)^. The findings revealed that these alterations may be attributed to preliminary changes in the vasculature as a result of risk factors, such as hypertensive or diabetic retinopathies^(^13^)^. In the present study, the percentage of individuals with systemic arterial hypertension was markedly higher in the group of patients affected by BRVO compared with the control group (81.3% versus 19.4%, respectively; p<0.001). This was expected, since hypertension is the principal risk factor for the development of this pathology^(^28^,^29^)^.

In the analysis of the subgroup with normal eyes, following adjustment for systemic arterial hypertension, there were no statistically significant differences found in any of the parameters evaluated by OCT angiography when this group was compared with the subgroup of contralateral eyes in patients with BRVO. These findings demonstrated that the differences between the normal and contralateral eyes may be due to early signs of hypertensive retinopathy.

Candidates for inclusion in the present study were screened to exclude any possible retinal pathology in the contralateral eye, except hypertensive retinopathy. Nevertheless, these results suggest that this test could be useful for detecting early changes that predispose to occlusion, many of which are currently imperceptible at fundoscopy, even if these early changes are a consequence of risk factors.

This was the first study conducted with treatment-naïve patients, highlighting the importance of examination at the initial consultation. The results show that macular edema, present in many of the patients affected by this disease, hampers the analysis of images and may lead to segmentation errors, particularly in the DCP of the retina. The ideal management would involve an individual analysis of each patient with respect to the segmentation of the superficial and deep layers of the retina.

The presence of macular edema hampers, but does not preclude, the use of density map parameters. However, an adequate evaluation of the image quality, possible artifacts, segmentation errors, and reliability indexes is essential.

In the present study, the whole image density and parafoveal density of the SCP and DCP were lower in the eyes affected by BRVO compared with those measured in the contralateral eyes and normal eyes. These parameters may represent an important tool for the diagnosis and follow-up of patients with BRVO. The values found are similar to those reported in previous studies. This contributes to establishing reference values (set points) for estimating the physiopathological damage caused by this disease. There were no statistically significant differences found between the eyes with respect to the foveal vascular density of the SCP and DCP. Therefore, it appears that this parameter is not suitable for the evaluation of this pathology.

Comparison of the contralateral eyes with the normal eyes revealed reductions in the same parameters. These reductions may be due to early changes in the vasculature as a consequence of risk factors, particularly systemic arterial hypertension. Nevertheless, these parameters are useful for evaluating early changes in eyes in which the risk of occlusion is greater. Further investigations are warranted to define the specific set points that would be indicative of a risk of occlusion.
